# Quantum Correlations and Coherence of Polar Symmetric Top Molecules in Pendular States

**DOI:** 10.1038/s41598-017-18148-6

**Published:** 2017-12-19

**Authors:** Zuo-Yuan Zhang, Jin-Ming Liu

**Affiliations:** State Key Laboratory of Precision Spectroscopy, East China Normal University, Shanghai, 200062 China

## Abstract

We consider two ultracold polar symmetric top molecules coupled by dipole-dipole interaction in an external electric field with appreciable intensity gradient, serving as the physical carrier of quantum information. Each molecule is induced to undergo pendular oscillations under the strong static electric field. Based on the pendular states of polar symmetric top molecules as candidate qubits, we investigate the bipartite quantum correlations of the two polar molecular system for the thermal equilibrium states, characterized by negativity and quantum discord, and then analyze the corresponding coherence, measured by relative entropy and *l*_1_ norm. Furthermore, we also examine the dynamics of the entanglement and coherence of the system in the presence of intrinsic decoherence, and explore the relations of their temporal evolution with various physical system parameters for two different initial Bell states. It is found that quantum correlations and coherence of the two polar molecules in pendular states can be manipulated by adjusting appropriate reduced variables including external electric field, dipole-dipole interaction, ambient temperature and decoherence factor. Our findings could be used for molecular quantum computing based on rotational states.

## Introduction

Quantum entanglement as an important physical resource in quantum information processing has attracted much attention since its discovery in 1935. It has a wide range of applications in many branches of physics, e.g., quantum teleportation^1,2^, quantum dense coding^3^, quantum key distribution^4^, and quantum computation^5–8^. By far, scientists have proposed several effective measures of entanglement, including relative entropy of entanglement^9,10^, entanglement of distillation^11^, entanglement of formation^12–14^ and negativity^15^.

But with the further development of quantum theory, people have found that the entanglement is not sufficient to describe all relations among subsystems. There still exist quantum correlation among some non-entangled states, i.e., non-entanglement does not necessarily mean classicality. In 2001, Ollivier and Zurek^16^ firstly introduced the concept of quantum discord (QD) to characterize the quantum correlation of the two-qubit states with von Neumann entropy, which can be used to detect some non-classical effects when the entanglement measures fail to do so. In recent years the quantum correlation plays key roles in some tasks of quantum communication and computation, and many theories^17–23^ and experiments^24–26^ about quantum correlations in various physical systems have gained wide attention.

As the core of interference phenomena, quantum coherence is a reflection of quantum superposition principle. It is also the necessary condition of quantum correlations. Recently, Baumgratz *et al*. put forward a rigorous framework to quantify coherence^27^, and defined two kinds of computable measures—relative entropy of coherence (REC) and *l*_1_ norm of coherence. Based on the definitions, the dynamics of coherence in open quantum system has been investigated^28,29^and some researches about the characteristics of coherence in quantum information process have been made^30–34^.

On the other hand, DeMille^35^ put forward a seminal proposal for physical implementation of a quantum computer by means of ultracold polar molecules. In DeMille’s proposal, diatomic molecules, trapped in a one-dimensional optical lattice and oriented along or against the external electric field, can entangle together due to the dipole-dipole interaction, with the polar molecular electric dipole moments employing as qubits. Meanwhile, rapid progresses have been made in various methods and techniques for producing, cooling and manipulating polar molecules in the past decades^36–39^. As a result, the trapped ultracold polar molecules as a carrier of quantum information were considered as a promising choice for quantum computation and simulation protocols^40–42^, because of polar molecules integrating the advantages of neural atoms and trapped ions.

Some people investigated the quantum entanglement and quantum computation of polar diatomic molecules in pendular states^43–47^, yet others examined the characteristics of rational states of polar molecules in electric, magnetic and optical fields^48–54^. However, to the best of our knowledge, seldom studies have involved with the quantum correlation and coherence of polar symmetric top molecules in pendular states. In this paper, we consider the model of two coupled symmetric top molecules in a static electric field with intensity gradient, and analyze the behavior of negativity, QD, REC and *l*_1_ norm of coherence for the two polar molecules in pendular states varying with the strength of external electric field, dipole-dipole interaction and ambient temperature. Moreover, we also study the temporal evolution of negativity and *l*_1_ norm of coherence of the two molecular system, and explore the relations of entanglement and coherence with decoherence factor and electric field strength for different initial states. We find that the negativity (characterizing entanglement) and QD (measuring quantum correlation) are negatively related to the electric field strength, and they are monotonically increasing functions with respect to the large dipole-dipole interaction. Furthermore, the quantum coherence (quantified by REC or *l*_1_ norm of coherence) of the molecular system is non-monotonic versus the intensity of electric field, and it is positively related to dipole-dipole interaction. Additionally, the high ambient temperature is always not favorable for the preservation of quantum entanglement, correlation and coherence. Our results show that the quantum correlations and coherence of the system can be manipulated by tuning the appropriate physical parameters and/or choosing proper initial states. These results may be useful to achieve the tasks of quantum information processing with molecular rotational states.

## Results

### The model

**Figure 1:**
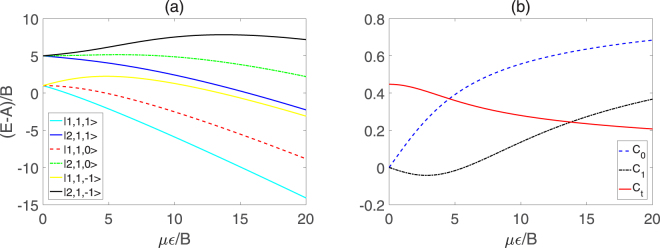
(**a**) Several sublevels of the symmetric top molecule in pendular states $|\tilde{J},K,{M}_{J}\rangle$ and (**b**) Molecular orientations *C*_0_, *C*_1_ and transition dipole moment *C*_*t*_, versus *με*/*B*.

For a trapped polar symmetric top molecule in an external electric field, the Hamiltonian can be given by

1 $$H=\frac{{{\boldsymbol{p}}}^{2}}{2m}+{V}_{trap}+B{{\boldsymbol{J}}}^{2}+(A-B){{\boldsymbol{J}}}_{z}^{2}-{\boldsymbol{\mu }}\cdot {\boldsymbol{\varepsilon }},$$

where *m* denotes the molecular mass, ***p*** ^2^/2*m* is the translational kinetic energy, *V*_*trap*_ is the potential energy, *A* and *B* denote the rotational constants, ***J*** is the total rotational angular momentum, ***J***_*z*_ is the projection on the external field direction for ***J***, ***μ*** is the body-fixed dipole moment, and ***ε*** is the electric field. Under the condition of ultracold temperature, the trapped molecule’s translational motion is very slow and nearly harmonic, thus the translational kinetic energy and potential energy can be omitted from the Hamiltonian. With the consideration of only retaining the rotational kinetic energy and the Stark energy, the Hamiltonian of the system^48^ is reduced to

2 $$H=B{{\boldsymbol{J}}}^{2}+(A-B){{\boldsymbol{J}}}_{z}^{2}-\mu \varepsilon \,\cos \,\theta ,$$

where *θ* denotes the angle between the dipole moment ***μ*** and the external electric field. It is easy to find that the rotation of molecule turns to procession about the field direction with the increase of field strength; if the field is strong enough, the Stark interaction will play a major role in Hamiltonian and the molecule will be compelled to undergo pendular oscillation instead of procession. The eigenenergy corresponding to the Hamilton *H* can be written as

3 $$E=BJ(J+1)+(A-B){K}^{2}+{E}_{S},$$

where *K* denotes the projection of the molecular angular momentum on inertia principal axes and *E*_*S*_ denotes the additional energy due to the Stark effect. In Fig. 1(a), we plot the sublevels of the polar symmetric top molecule in pendular states for $\tilde{J}=1$ or 2, and *K* = 1 as a function of *με*/*B*. Here, we choose the *M*_*J*_ = 0, *K* = 1 pendular states with $\tilde{J}=1$ and 2 as the qubits |0〉 and |1〉, respectively, where *M*_*J*_ is the projection of the molecular angular momentum on the direction of external electric field. However, $\tilde{J}$ is not a good quantum number anymore because of the mixture of Stark effect and the rotational states, whereas *M*_*J*_ maintains good. Due to the completeness of the Hilbert space consisting of base vectors $\sum |J,K,{M}_{J}\rangle$, the pendular qubit states chosen can be given by the superposition of rotational states $|J,K,{M}_{J}\rangle$ ^55^,

4 $$|0\rangle =\sum _{J}{a}_{J}|J,K,{M}_{J}\rangle ,|1\rangle =\sum _{J}{b}_{J}|J,K,{M}_{J}\rangle .$$

**Figure 2:**
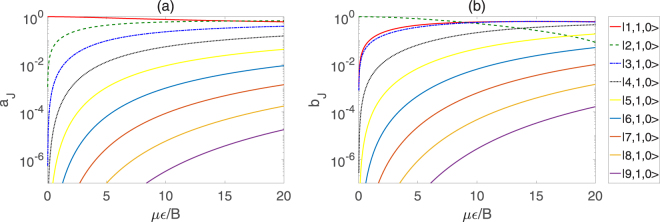
Coefficients *a*_*J*_ and *b*_*J*_ of the sum of $|J,K,{M}_{J}\rangle$ for qubits |0〉 and |1〉 as a function of *με*/*B*, respectively.

Figure 2 depicts the coefficients *a*_*J*_ and *b*_*J*_ for pendular states |0〉 and |1〉 as a function of *με*/*B* respectively. It can be observed from Fig. 2(a) that, as we expect, the coefficient *a*_1_ of |1, 1, 0〉 is 1 while all other coefficients *a*_*J*_ (*J* = 2, 3, …, 9) is 0 in the absence of the external electric field, and with the increase of the electric field strength, the coefficient *a*_1_ decreases gradually, whereas the proportions of the other coefficients becomes larger. Moreover, we can learn from Fig. 2(b) that the coefficient *b*_2_ of |2, 1, 0〉 decreases gradually from 1 with the electric field strength increasing, and when *με*/*B* increases to respective critical values, the other coefficients may be greater than the corresponding *b*_2_ of |2, 1, 0〉.

The orientations *C*_0_ and *C*_1_ are defined by the expectation values of cos *θ* under the basis of the pendular states |0〉 and |1〉 respectively, who determine the effective dipole moments of the molecules. The transition dipole moment between the two pendular states is set to be *C*_*t*_. They are given by

5 $${C}_{0}=\langle 0|\cos \,\theta |0\rangle ,{C}_{1}=\langle 1|\cos \,\theta |1\rangle ,{C}_{t}=\langle 0|\cos \,\theta |1\rangle .$$

Figure 1(b) shows that, with *με*/*B* increasing, the effective dipole moment *C*_0_ increases monotonously from 0 whereas the transition dipole moment *C*_*t*_ is reduced from 0.447. Moreover, the other effective dipole moment *C*_1_ first diminishes to a minimal value and then increases gradually as *με*/*B* grows.

Let us consider two identical polar symmetric top molecules, the dipole-dipole coupling interaction between the two molecules can be given by

6 $${V}_{d-d}=\frac{{{\boldsymbol{\mu }}}_{1}\cdot {{\boldsymbol{\mu }}}_{2}-3({{\boldsymbol{\mu }}}_{1}\cdot {\boldsymbol{n}})({{\boldsymbol{\mu }}}_{2}\cdot {\boldsymbol{n}})}{{r}_{12}^{3}}.$$

Here ***μ***_*x*_ (*x* = 1, 2) denote the dipole moments of the molecules in different positions, *r*_12_ = |***r***_1_ − ***r***_2_| denotes the distance between the two molecules, ***n*** represents a unit vector along ***r***_12_. When the interaction between the polar molecules and the electric field is stronger than the dipole-dipole coupling, the effective dipole-dipole interaction can be approximately simplified through averaging the azimuthal angles^48,56^ to

7 $${V}_{d-d}={\rm{\Omega }}(1-3{\cos }^{2}\alpha )\cos \,{\theta }_{1}\,\cos \,{\theta }_{2},$$

where ${\rm{\Omega }}={\mu }^{2}/{r}_{12}^{3}$, *θ*_*x*_ denotes the angle between the dipole moment ***μ***_*x*_ and the external electric field, and *α* denotes the angle between ***r***_12_ and the external electric field.

It is worth mentioning that, to make the molecular qubits addressable effectively, the electric field gradient difference is assumed to be 0.01 throughout this paper. Because of the gradient difference, the interaction energy is different between every molecular dipole moment and the external field, which leads to the difference of every molecular electric-dipole transition frequency, thereby it is convenient for us to choose an appropriate microwave fields for addressing the molecular quantum qubits individually.

If taking the pendular states {|00〉, |01〉, |10〉, |11〉} as a set of basis vectors, the Hamiltonian of two coupled molecules due to the dipole-dipole interaction takes the following form:

8 $$H^{\prime} ={H}_{1}+{H}_{2}+{V}_{d-d},$$

where

$${H}_{1}=(\begin{array}{cc}{E}_{0}^{1} & 0\\ 0 & {E}_{1}^{1}\end{array})\otimes I,{H}_{2}=I\otimes (\begin{array}{cc}{E}_{0}^{2} & 0\\ 0 & {E}_{1}^{2}\end{array}),{V}_{d-d}={\rm{\Omega }}(\begin{array}{cc}{C}_{0}^{1} & {C}_{t}^{1}\\ {C}_{t}^{1} & {C}_{1}^{1}\end{array})\otimes (\begin{array}{cc}{C}_{0}^{2} & {C}_{t}^{2}\\ {C}_{t}^{2} & {C}_{1}^{2}\end{array}).$$

Here, ${E}_{0}^{x}$ and ${E}_{1}^{x}(x=\mathrm{1,}\,2)$ denote the eigenenergies of pendular states |0〉 and |1〉, respectively, *I* is a two-dimensional identity matrix, as well as ${C}_{0}^{x}$, ${C}_{1}^{x}$ and ${C}_{t}^{x}$ are orientations and transition dipole moment mentioned above.

In order to calculate the quantum correlations and coherence of the system consisting of two polar symmetric top molecules in the thermal equilibrium states, we introduce a density matrix involving temperature, *ρ*(*T*) = exp(−*βH*′)/*Z*(*T*), with *β* = 1/(*k*_*B*_ *T*), where *k*_*B*_ is the Boltzmann constant, *Z*(*T*) is the partition function given by

9 $$Z(T)=Tr[\exp (-\beta H^{\prime} )]=\sum _{i}{e}^{-\beta {E}_{i}},$$

where *E*_*i*_ denotes the *i*th eigenvalue of the Hamiltonian *H*′. Then the density matrix can be rewritten as

10 $$\rho (T)=\frac{1}{Z}\sum _{i=1}^{4}{e}^{-\beta {E}_{i}}|{\psi }_{i}\rangle \langle {\psi }_{i}|,$$

where |*ψ*_*i*_〉 is the *i* th eigenfunction corresponding to the eigenvalue *E*_*i*_. Based on this density matrix *ρ*(*T*), we can evaluate the quantum correlations and coherence of the two symmetric top molecules.

### Quantum correlations and coherence in thermal equilibrium states

Herein, we turn to investigate the evolution property of negativity, QD and coherence with respect to the functions of *με*/*B*, Ω/*B* and *k*_*B*_ *T*/*B*. For simplicity, in the following the electric field is supposed to be perpendicular to the direction of ***r***_12_, i.e., *α* = *π*/2.

**Figure 3:**
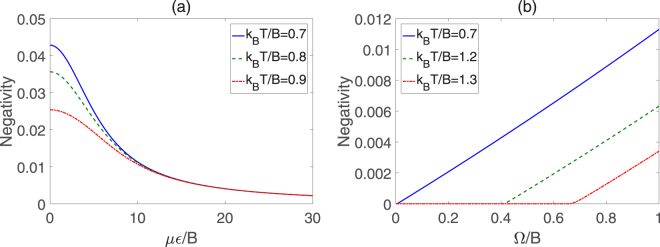
Negativity of two symmetric top molecules in pendular states for different *k*_*B*_ *T*/*B*. (**a**) Negativity versus *με*/*B* with Ω/*B* = 1, (**b**) Negativity versus Ω/*B* with *με*/*B* = 10.

**Figure 4:**
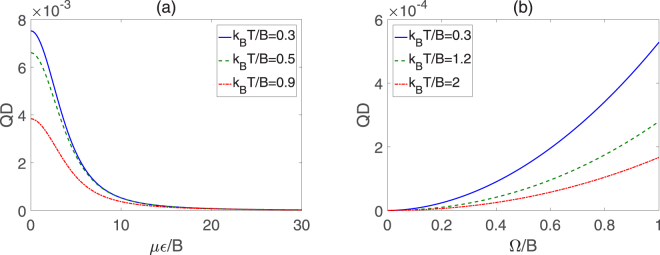
QD of two symmetric top molecules in pendular states for different *k*_*B*_ *T*/*B*. (**a**) QD versus *με*/*B* with Ω/*B* = 1, (**b**) QD versus Ω/*B* with *με*/*B* = 10.

Through numerical calculations, we depict the variation behavior of negativity versus *με*/*B* and Ω/*B* for different *k*_*B*_ *T*/*B* in Fig. 3. We can see from Fig. 3(a) that the negativity decreases monotonously with the increase of *με*/*B* for different values of temperature, and when *με*/*B* is large, the entanglement becomes very weak. In the weak electric field, the negativity is inversely related to the temperature. However, when *με*/*B* > 13, the negativities are almost equal for different *k*_*B*_ *T*/*B*, which means that the entanglement of the molecular system hardly depends on the temperature if the electric field is strong enough. We can observe from Fig. 3(b) that at the high temperature (*k*_*B*_ *T*/*B* = 1.2 or 1.3), the negativity is nearly nonexistent if the dipole-dipole interaction is very weak. But in the scope of the strong dipole-dipole interaction, it is obvious that the negativity increases monotonously with the enhancing of Ω/*B*. Moreover, at the same value of Ω/*B*, the larger *k*_*B*_ *T*/*B* is, the smaller the negativity is. Thus, in order to obtain more entanglement resource, the environment temperature should decrease as far as possible. Figure 4 displays the QD as a function of *με*/*B* and Ω/*B*, in which there exist remarkable negative relation for QD versus *με*/*B* or *k*_*B*_ *T*/*B*, similar to the previous cases of negativity. Additionally, QD exhibits a nonlinear increase with the growth of Ω/*B*, as shown in Fig. 4(b). As we know, the entanglement can be regarded as a kind of quantum correlation, so the negativity and QD follow the similar evolutionary trends to some extent.

**Figure 5:**
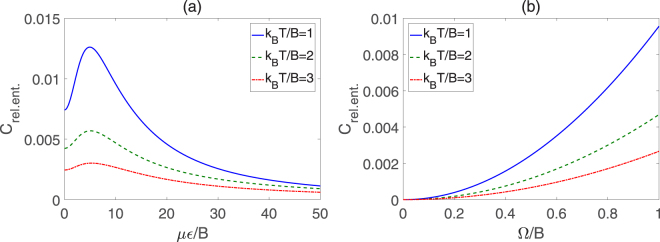
REC for two symmetric top molecules in pendular states for different *k*_*B*_ *T*/*B*. (**a**) Coherence versus *με*/*B* with Ω/*B* = 1, (**b**) Coherence versus Ω/*B* with *με*/*B* = 10.

**Figure 6:**
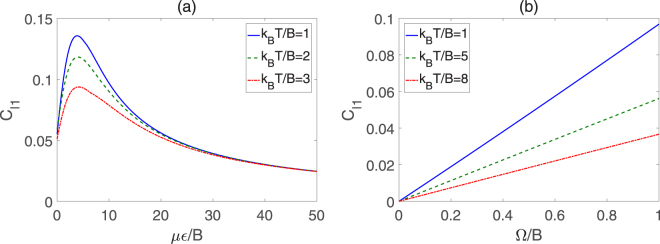
*l*_1_ norm of coherence for two symmetric top molecules in pendular states for different *k*_*B*_ *T*/*B*. (**a**) Coherence versus *με*/*B* with Ω/*B* = 1, (**b**) Coherence versus Ω/*B* with *με*/*B* = 10.

Figure 5 plots the variations of REC with *με*/*B* and Ω/*B* for *k*_*B*_ *T*/*B* = 1, 2 and 3. It can be observed from Fig. 5(a) that the coherence shows an increase in pre-peak then occurs a gradual decline in the trend with the increase of *με*/*B*. From Fig. 5(b), we can find that the REC increases monotonously with Ω/*B* growing. Moreover, the REC decreases with the increase of *k*_*B*_ *T*/*B* when other parameters are fixed, which implies that the coherence of the molecular system suffers from attenuation as the external temperature increases. Figure 6 shows the *l*_1_ norm of coherence as a function of *με*/*B* and Ω/*B* for different temperature. It can be seen that the *l*_1_ norm of coherence has the similar variance tendency as the REC. Thus, the two kinds of methods for quantifying coherence are almost equivalent to some degree.

### Influence of intrinsic decoherence on entanglement and coherence

Next, we consider the effect of intrinsic decoherence on negativity and *l*_1_ norm of coherence of the two coupled symmetric top molecules by analytically solving Milburn’s equation^57^ for the case of two different initial Bell states^48^, and analyze the relations of temporal evolution with the decoherence factor *γ* and *με*/*B*.

**Figure 7:**
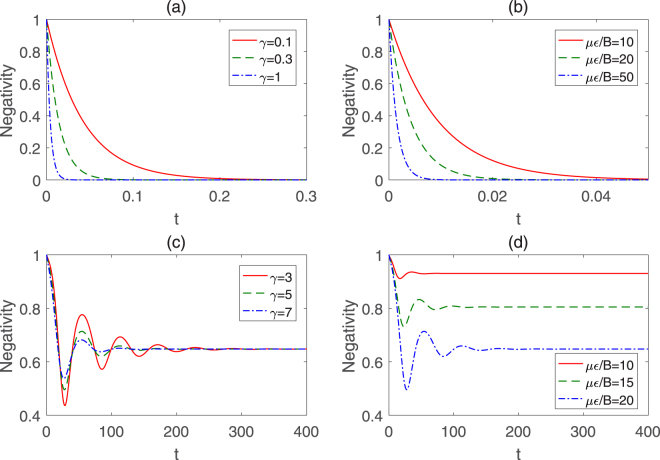
Temporal evolutions of the negativity with Ω/*B* = 1 for two kinds of different initial states. The initial Bell state is in the form of $(|00\rangle +|11\rangle )/\sqrt{2}$ for (**a**) different *γ* with *με*/*B* = 20 and (**b**) different *με*/*B* with *γ* = 1, the initial Bell state is in the form of $(|01\rangle +|10\rangle )/\sqrt{2}$ for (**c**) different *γ* with *με*/*B* = 20 and (**d**) different *με*/*B* with *γ* = 5.

Figure 7(a) shows that the negativity decays exponentially over time for the initial Bell state $(|00\rangle +|11\rangle )/\sqrt{2}$. When *t* = 0, the negativity is 1 since the initial state is maximally entangled before interacting with the environment. After the evolution for a period of time, the entanglement approximates to 0. Additionally, it is found that the larger decoherence factor can lead the negativity to decrease more rapidly, the reason is that larger decoherence factor implies stronger coupling between the symmetric top molecules system and external environment. Figure 7(b) depicts the relation of temporal evolution with different *με*/*B* for *γ* = 1. We can see that for the same time *t*, the larger *με*/*B* is, the smaller the negativity is, which means that the strong external field can accelerate the process of decoherence for a given symmetric top molecules system.

As seen from Fig. 7(c), for the initial state $(|01\rangle +|10\rangle )/\sqrt{2}$, the negativity undergoes some damped oscillations and approaches to a constant after long enough time, which implies that the entanglement of this Bell state is more robust in intrinsic decoherence evolution compared with the situation of Fig. 7(a). Besides, we can also find that the decay rate is positive correlated with the decoherence factor, but the periods of oscillations are identical for different *γ*. It can be explained qualitatively from the exponential term in equation (20). Specifically, the parameter *γ* in the term $\exp \,[-\frac{\gamma t}{2}{({E}_{m}-{E}_{n})}^{2}]$ affects the decay rate whereas the oscillation frequency only depends on the term exp[−*it*(*E*_*m*_−*E*_*n*_)]. We plot the influence of the electric field strength on the temporal evolution for the initial state $(|01\rangle +|10\rangle )/\sqrt{2}$ in Fig. 7(d). From the sub-figure we can see that the stronger electric field results in more numbers of oscillations during the evolution and smaller entanglement after evolution time. Thus, we can obtain the ampler and more stable entanglement resource in this Bell state by adopting the weak electric field, which is remarkable different from the cases for the initial state $(|00\rangle +|11\rangle )/\sqrt{2}$, whereas the entanglement almost decays to 0 eventually in an asymptotical way (see Fig. 7(a) and (b)).

**Figure 8:**
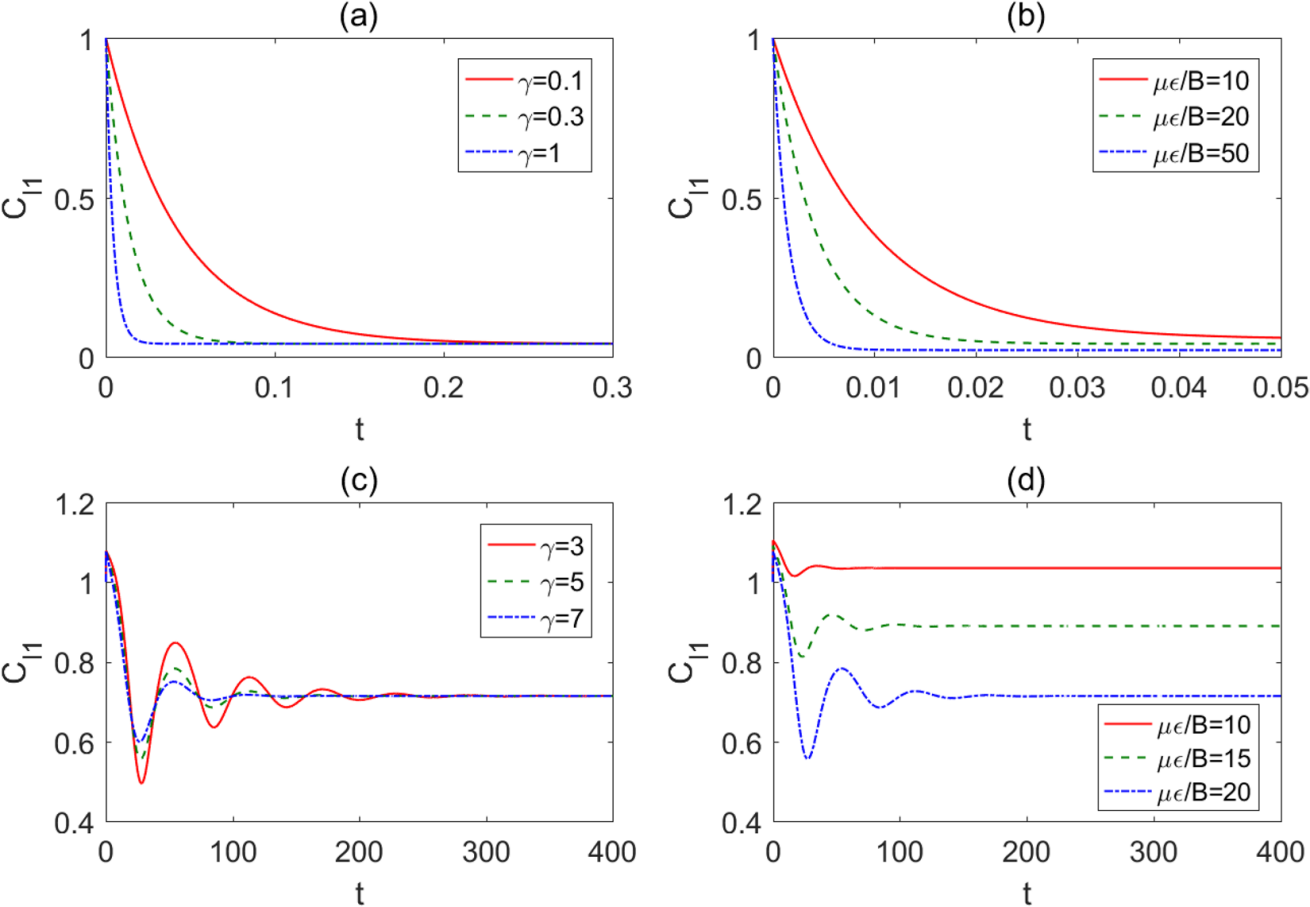
Temporal evolutions of the *l*_1_ norm of coherence with Ω/*B* = 1 for two kinds of different initial states. The initial Bell state is in the form of $(|00\rangle +|11\rangle )/\sqrt{2}$ for (**a**) different *γ* with *με*/*B* = 20 and (**b**) different *με*/*B* with *γ* = 1, the initial Bell state is in the form of $(|01\rangle +|10\rangle )/\sqrt{2}$ for (**c**) different *γ* with *με*/*B* = 20 and (**d**) different *με*/*B* with *γ* = 5.

Next, we investigate the behavior of the temporal evolution of *l*_1_ norm of coherence under the same conditions as the negativity. It can be seen from Figs 7 and 8 that the variance tendencies of coherence and entanglement are almost the identical except for the evolution amplitudes. As plotted in Fig. 8(a) and (b), the *l*_1_ norm of coherence decays monotonously with the time for the initial Bell state $(|00\rangle +|11\rangle )/\sqrt{2}$, and the decoherence factor or the strength of the electric field has negative impacts on the preservation of coherence. However, the *l*_1_ norm of coherence does not vanish eventually but evolve to a nonzero value asymptotically, which implies that the coherence of the initial state $(|00\rangle +|11\rangle )/\sqrt{2}$ still exists after enough time while the entanglement nearly disappears (see Fig. 7(a) and (b)). Moreover, Fig. 8(c) shows that the coherence first increases rapidly from 1 and then decays with oscillations to a stable value for the case of the initial Bell state $(|01\rangle +|10\rangle )/\sqrt{2}$. As seen from Fig. 8(d), the *l*_1_ norm of coherence will surpass 1 in a long time limit when *με*/*B* = 10, in comparison with Fig. 7(d), which indicates that the steady coherence may be larger than the initial one while the entanglement can not exhibit the novel phenomenon.

## Discussion

In summary, by selecting the pendular states |1, 1, 0〉 and |2, 1, 0〉 consisting of the rotational states of the symmetric top molecules in an external electric field as candidate qubits, we have investigated the quantum entanglement, correlation and coherence of the two identical polar symmetric top molecules coupled by dipole-dipole interaction. After numerically calculating the negativity, QD, REC, and *l*_1_ norm of coherence of the molecular system in the thermal equilibrium states, we have analyzed their relations with three dimensionless reduced parameters including the electric field strength, dipole-dipole interaction, and temperature. Our findings are listed as follows. Firstly, both the negativity and the QD decrease with the growth of *με*/*B*, which implies that the quantum entanglement and correlation are negatively related with the external electric field. This is because the Stark interaction between the external electric field and the polar molecule plays a major role with the electric field enhancing, whereas the interactions between pendular states decrease relatively. However, the REC and the *l*_1_ norm of coherence grow to the peak value firstly and then decrease gradually as *με*/*B* increases, which indicates clearly that the behavior of the coherence is not synchronous completely with that of the quantum entanglement or correlation. Secondly, the negativity, QD and coherence display general trends of increase as functions of dipole-dipole interaction, i.e., they can be reinforced by strengthening the dipole-dipole coupling. Thirdly, the temperature has negative impact on the quantum entanglement, correlation and coherence. This maybe lie in that the molecules in rotational ground states are excited to the ro-vibrational states in a higher energy level with more probability as the temperature increases, and the decrease of the population in ground states weakens the interactions between pendular states. In terms of above analyses, the quantum correlations and coherence of the two coupled symmetric top molecules in pendular states can be manipulated by adjusting the strength of the external field, dipole-dipole interaction and ambient temperature.

On the other hand, we have also examined the intrinsic decoherence effects on the quantum entanglement and coherence of the system for two different initial Bell states by solving Milburn’s equation. It is found that the small decoherence factor and weak electric field are helpful to suppress the decay of the negativity and coherence in the process of temporal evolution. For the initial state $(|00\rangle +|11\rangle )/\sqrt{2}$, the quantum entanglement and coherence are easier to be destroyed by the external environment and electric field, in contrast to the cases of initial state $(|01\rangle +|10\rangle )/\sqrt{2}$. Thus, the Bell state $(|01\rangle +|10\rangle )/\sqrt{2}$ is more robust for the molecular system against the intrinsic noise, and could be considered as a better choice for achieving much amount of entanglement or coherence.

The recent developments in manufacturing, cooling and trapping molecules provide opportunities to choose experimentally the polar molecules as the carrier for quantum information processing. However, the dipole-dipole interaction is very small in practice (Ω < 10^−5^ in typical optical lattice), which is far from meeting the requirement of the optimal quantum correlations and coherence mentioned in our paper. Fortunately, the intermolecular distance can be shortened to about 10 nm by exploiting the nanoscale plasmon-enhanced electric or electro-optical traps reported in the literatures^58,59^, which may be helpful to strengthen the dipole-dipole interaction to some extent. Actually, reducing the distance so dramatically would foster the spontaneous Raman scattering of lattice photons, which could result in further decoherence. Hence, moderate intermolecular distance would be a critical factor to be considered in experiment. In addition, it deserves emphasizing that considering the limitation of practical experimental conditions, the electric field strength is usually restricted in a finite range. In this way, the symmetric top molecules we studied can not be in pendular states strictly but correspond to an intermediate cases in the weak electric field. We expect that our findings might strengthen the understandings of the relations among quantum entanglement, correlation and coherence, and could stimulate the further studies on quantum nonlocality in molecular systems.

## Methods

### Negativity

Based on Peres’ criterion for separability, Vidal and Werner^15^ proposed a novel computable measure of entanglement—negativity, which is defined as

11 $$N(\rho )=\sum _{i}|{\lambda }_{i}({\rho }^{{T}_{A}})|-\mathrm{1,}$$

where *ρ* is the density matrix of a bipartite mixed state, ${\lambda }_{i}({\rho }^{{T}_{A}})$ is the *i*th eigenvalue of the partial transpose matrix ${\rho }^{{T}_{A}}$ corresponding to the subsystem *A*. The negativity *N* (*ρ*) has been proved not to increase under local operations and classical communication, and can be used to measure entanglement of a bipartite system.

### Quantum discord

In quantum information theory, the total correlation for bipartite states can be measured by the mutual information:

12 $$I({\rho }^{AB})=S({\rho }^{A})+S({\rho }^{B})-S({\rho }^{AB}),$$

where *I* denotes the mutual information, *ρ* ^*AB*^ is the density matrix of the bipartite quantum system, *ρ* ^*A*^ and *ρ* ^*B*^ are reduced density matrices of the subsystems A and B, respectively, and $S(\rho )=-Tr(\rho {\mathrm{log}}_{2}\rho )$ is von Neumann entropy. The classical correlation of the quantum system can be defined as

13 $${J}_{A}({\rho }^{AB})=S({\rho }^{B})-S({\rho }^{AB}|\{{\Pi }_{k}^{A}\}).$$

Here, $S({\rho }^{AB}|[{\Pi }_{k}^{A}])$ is the conditional entropy with a POVM $\{{\Pi }_{k}^{A}\}$ acting on the subsystem *A*, and *J*_*A*_ is the classical correlation varying with different measurement basis vectors. In order to make the quantum correlation independent of measurement basis vectors, *J*_*A*_ should take the maximum under all possible POVM. The quantum discord^16^ is defined as the difference between the total correlation and the maximal classical correlation:

14 $${D}_{A}({\rho }^{AB})=I({\rho }^{AB})-\mathop{max}\limits_{\{{\Pi }_{k}^{A}\}}{J}_{A}({\rho }^{AB}).$$

### Coherence

Baumgratz *et al*.^27^ proposed two measures to evaluate the coherence of a quantum system. One is relative entropy of coherence (REC), which is defined by the minimal distance between the arbitrary quantum state *ρ* and the incoherent state *δ*:

15 $${C}_{rel.ent.}=\mathop{{\rm{\min }}\,}\limits_{\delta \in I}S(\hat{\rho }||\hat{\delta })=S({\hat{\rho }}_{diag})-S(\hat{\rho }),$$

where $\hat{\delta }$ is the incoherent state belonging to the collection *I* of all incoherent states, ${\hat{\rho }}_{diag}$ is the density matrix obtained by deleting all the off-diagonal elements of the arbitrary state *ρ*, and $S(\rho )=-Tr(\rho {log}_{2}\rho )$ is the von Neumann entropy. The other is *l*_1_ norm of coherence given by the summation of all off-diagonal elements’ absolute values as follows

16 $${C}_{l1}(\hat{\rho })=\sum _{i,j,i\ne j}|{\rho }_{i,j}|.$$

Generally speaking, ${C}_{l1}(\hat{\rho })$ is more essential to measure the coherence of quantum system and easier to be calculated in contrast to the REC. Both definitions meet all requirements for a reasonable quantum coherence measure. In this paper, we adopt the two methods mentioned above to measure the coherence of two coupled polar symmetric top molecular system within the thermal equilibrium environment and investigate the temporal evolution of *l*_1_ norm of coherence in the presence of intrinsic decoherence.

### Intrinsic decoherence

Generally, a realistic physical system always interacts with its external environment, which will result in the loss of the coherence of this system. To investigate the time evolution of the molecular system, we adopt the Milburn model^57^, which is a simple modification for Schrödinger equation by introducing a decoherence factor to describe the phase decoherence in Markov environment. According to Milburn’s equation, the density matrix of the quantum system evolving with time is defined as

17 $$\frac{d}{dt}\rho (t)=\frac{1}{\gamma }[\exp (-i\gamma H)\rho (t)\exp (i\gamma H)-\rho (t)],$$

where *γ* is the decoherence factor, *H* is the Hamilton of quantum system. Under the approximate Markovian condition, equation (17) can be rewritten as

18 $$\frac{d}{dt}\rho (t)=-i[H,\rho (t)]-\frac{\gamma }{2}[H,[H,\rho (t)]],$$

the solution of above equation is given by

19 $$\rho (t)=\mathop{\sum _{k=0}}\limits^{{\rm{\infty }}}\frac{{(\gamma t)}^{k}}{k!}{H}^{k}\exp (-iHt)\exp (-\frac{\gamma t}{2}{H}^{2})\rho (0)\exp (-\frac{\gamma t}{2}{H}^{2})\exp (iHt){H}^{k},$$

where *ρ*(0) is the initial density operator of the quantum system. If setting *E*_*i*_ and $|{\psi }_{i}\rangle (i=m,n)$ as the *i*th eigenvalues and eigenstates of the Hamilton respectively, the density matrix can be given by

20 $$\rho (t)=\sum _{m,n}\exp [-\frac{\gamma t}{2}{({E}_{m}-{E}_{n})}^{2}-i({E}_{m}-{E}_{n})t]\times \langle {\psi }_{m}|\rho (0)|{\psi }_{n}\rangle |{\psi }_{m}\rangle \langle {\psi }_{n}|.$$

Based on above equation, we can investigate the influence of intrinsic decoherence on the temporal evolution of entanglement and coherence for the initial quantum state *ρ*(0).
